# Overexpressing the Multiple-Stress Responsive Gene At1g74450 Reduces Plant Height and Male Fertility in *Arabidopsis thaliana*

**DOI:** 10.1371/journal.pone.0140368

**Published:** 2015-10-20

**Authors:** Anne M. Visscher, Eric J. Belfield, Daniela Vlad, Niloufer Irani, Ian Moore, Nicholas P. Harberd

**Affiliations:** Department of Plant Sciences, University of Oxford, Oxford, OX1 3RB, Oxfordshire, United Kingdom; Indiana University, UNITED STATES

## Abstract

A subset of genes in *Arabidopsis thaliana* is known to be up-regulated in response to a wide range of different environmental stress factors. However, not all of these genes are characterized as yet with respect to their functions. In this study, we used transgenic knockout, overexpression and reporter gene approaches to try to elucidate the biological roles of five unknown multiple-stress responsive genes in Arabidopsis. The selected genes have the following locus identifiers: At1g18740, At1g74450, At4g27652, At4g29780 and At5g12010. Firstly, T-DNA insertion knockout lines were identified for each locus and screened for altered phenotypes. None of the lines were found to be visually different from wildtype Col-0. Secondly, 35S-driven overexpression lines were generated for each open reading frame. Analysis of these transgenic lines showed altered phenotypes for lines overexpressing the At1g74450 ORF. Plants overexpressing the multiple-stress responsive gene At1g74450 are stunted in height and have reduced male fertility. Alexander staining of anthers from flowers at developmental stage 12–13 showed either an absence or a reduction in viable pollen compared to wildtype Col-0 and At1g74450 knockout lines. Interestingly, the effects of stress on crop productivity are most severe at developmental stages such as male gametophyte development. However, the molecular factors and regulatory networks underlying environmental stress-induced male gametophytic alterations are still largely unknown. Our results indicate that the At1g74450 gene provides a potential link between multiple environmental stresses, plant height and pollen development. In addition, ruthenium red staining analysis showed that At1g74450 may affect the composition of the inner seed coat mucilage layer. Finally, C-terminal GFP fusion proteins for At1g74450 were shown to localise to the cytosol.

## Introduction

As sessile organisms, plants depend on their ability to coordinate growth and development with environmental conditions [1]. Stress in plants can be defined as an altered physiological state caused by factors that tend to change equilibrium, resulting in injury, disease or aberrant physiology in the case of a negative (distress) effect [2]. The balance between tolerance and sensitivity in particular plant species may determine whether a stress factor has positive (eustress) or negative effects [3]. In model plants like *Arabidopsis thaliana*, tolerance is often measured as “survival”, whilst in crop species like cereals, maintenance of “yield” and “productivity” is for economic reasons more important than survival [4]. The shifting of breeding goals towards “low input, high output” agriculture increases the need for genetic dissection of quantitative traits controlling adaptive responses and performance of crops under environmentally constrained conditions, especially during developmental stages such as male gametophyte development, the transition to flowering phase or grain filling [5].

Whilst some stress regulated genes in plants are differentially expressed in response to particular stress factors, others react to a broad spectrum of adverse environmental conditions. For example, colleagues identified a cluster of 197 genes in Arabidopsis that are induced by a wide range of diverse stress conditions, such as cold, osmotic shock, salinity, wounding and biotic stress factors (including treatments with elicitors) [6]. According to the authors, these 197 genes appear to represent a common or universal stress response transcriptome, since many of the genes are conserved among plants, animals and fungi and are stress regulated in all organisms. The timing of expression of these genes can be early: within 45 minutes of exposure to abiotic stress, the expression of several multiple-stress responsive genes in Arabidopsis was found to be significantly up-regulated [7].

Although many of the 197 common stress genes are known to be involved in processes ranging from MAPK and Snf1 activities to ER stress, vesicle transport, phospholipid signalling, ROS, Ca^2+^, ABA, mitochondrial function and transcription, 35 out of the original 40 unknown genes in this set are still not characterized with respect to the biological and biochemical processes in which their protein products are engaged [6]. To put this in context, approximately 40% of Arabidopsis and 1% of rice (*Oryza sativa*) protein-coding genes have had some aspect of their functions annotated based on experimental evidence [8]. However, only about 5% of Arabidopsis genes are experimentally characterized for all of Gene Ontology’s (GO) three functional domains, which include biological process, cellular compartment and molecular function [8].

Reverse genetic approaches such as transgenic knockout, overexpression and reporter gene analyses may help determine the functions of the remaining uncharacterized common stress-responsive genes in Arabidopsis. For the current study, five multiple-stress responsive genes of unknown function were chosen based on available knockout lines. In addition, overexpressing lines were constructed for each of the five sequences. The selected genes have the following locus identifiers: At1g18740, At1g74450, At4g27652, At4g29780 and At5g12010. The amino acid sequences corresponding to these loci are 70% identical for At18740 and At1g74450, whilst a sequence similarity of 59% is found in the case of At4g29780 and At5g12010. Despite the fact that there is no experimentally based annotation available regarding the biological and molecular functions of these (related) genes, large-scale sequence analysis, gene expression and protein (interaction) studies have generated some information that is summarized below.

Experimental evidence suggests that the At5g12010 protein is localised to the plasma membrane and the membrane of various cellular compartments, including the vacuole [9–11]. Based on computational analyses, the protein is predicted to be involved in proline transport [12]. Several comprehensive studies using transgenic plants or mutants have demonstrated that proline metabolism has a complex effect on development and stress responses, and that proline accumulation is important for the tolerance of certain adverse environmental conditions [13].

Yeast two-hybrid studies have indicated that the At4g29780 protein, which is 59% identical to At5g120120, shows interaction with N-myc Downregulated-Like 1 (NDL1) and Regulator of G-protein Signaling 1 (RGS1) proteins [14]. However, these findings are not yet confirmed *in planta*. Arabidopsis NDL1 interacts with RGS1 as part of a multimeric protein complex to regulate auxin transport at the membrane and, consequently, the formation of lateral roots [15]. Heavy metals, nutrient deficiencies and hypoxia are all known to change the formation and/or elongation of lateral roots and auxin is thought to play a key role in these morphogenic stress responses [16].

Whilst no experimental evidence exists regarding the three functional GO domains of the At4g27652, At1g18740 and At1g74450 proteins, analysis of large-scale transcriptomic data shows that At1g74450 is most highly expressed in mature pollen [17–19]. Given the fact that the expression of the five genes described above is up-regulated in response to a variety of abiotic and biotic stresses, our study investigated whether their protein products play functionally important roles in stress resilience, growth or reproductive remodelling, as evidenced by significant phenotypical changes in knockout and overexpression lines.

## Materials and Methods

### Knockout mutant line confirmation

In order to identify potential knockout lines for the five intronless genes that were selected for this study, the following SALK T-DNA insertion lines were ordered from the Nottingham Arabidopsis Stock Centre (NASC): SALK_020993 (At1g18740), SALK_145820 (At1g74450), SALK_071404 (At4g27652), SALK_088755 (At4g29780) and SALK_024636 (At5g12010) [20]. For backcrossing and knockout line comparisons to wildtype, the Arabidopsis background wildtype line that was used to generate the SALK T-DNA stocks [20] was also ordered from NASC. This line, which carried NASC stock number N60000, is currently referred to as Col-0 but was formerly listed as Col-8.

The locations of the exonic T-DNA insertions were confirmed by PCR using MangoTaq DNA Polymerase and dNTPs (Bioline). Genomic DNA was extracted from N60000 (Col-0) and mutant leaves according to the Shorty method described previously [7]. S1 Table lists the left and right genomic primers that were chosen for each line based on outputs from the T-DNA primer design programme (http://signal.salk.edu/tdnaprimers.2.html). In addition, the left T-DNA border primer LBb1.3 was used in combination with each right genomic primer to detect the T-DNA insertions.

Absence of relevant mRNA in each line was confirmed by RT-PCR (OneStep RT-PCR Kit, Qiagen). RNA was extracted from Col-0 and homozygous mutant leaves with the RNeasy Plant Mini Kit (Qiagen) and treated with RNase-Free DNase (Qiagen).

Gene specific primers were chosen based on genomic DNA sequence information (NCBI) and outputs from the Primer3 programme. The selected left and right primers for each T-DNA insertion line are listed in S1 Table. Alpha-(α)-Tubulin was used as a constitutive control with primers as published previously [21].

The confirmed knockout lines were backcrossed to Col-0 four times prior to self-fertilization and selection of homozygous knockout lines. Subsequently, a double knockout line was created by crossing single knockout lines for the related At1g18740 and At1g74450 genes.

### Transgenic construct generation

As all five genes are intronless, genomic DNA extracted from Col-0 leaves with the DNeasy Plant Mini Kit (Qiagen) was used as a template for the cloning of open reading frames (ORFs) and promoter sequences. At1g18740 and At1g74450 ORFs were amplified by PCR for blunt-end TOPO cloning into the pENTR TOPO vector (Invitrogen) using PfuTurbo DNA polymerase AD (Agilent Technologies). At4g27652, At4g29780 and At5g12010 ORFs, as well as promoter sequences for all five genes, were amplified by PCR for *att*B site recombination with the pDONR207 entry vector (Invitrogen) using Phusion High-Fidelity DNA Polymerase (Finnzymes, Thermo Fisher Scientific). The selected promoter sequences were approximately 2,000 bases upstream of the protein coding sequences: At1g18740 (1,866 bp), At1g74450 (2,331 bp), At4g27652 (2,007 bp), At4g29780 (2,169 bp) and At5g12010 (2,751 bp).

N-terminal fusion primers (with stop codon) and C-terminal fusion primers (without stop codon) were designed based on coding or full chromosome sequences (NCBI) and are listed in S1 Table. PCR products were extracted from agarose gels with the QIAquick Gel Extraction Kit (Qiagen). Following this, the blunt-end PCR products were incorporated into the pENTR TOPO vector, whilst the *att*B PCR products were recombined with the pDONR207 entry vector using BP Clonase II Enzyme Mix (Invitrogen). Subsequently, DH5α competent *Escherichia coli* (*E*. *coli*) cells were transformed with the created entry clones using heat shock. Transformed colonies were grown overnight on selective media and confirmed by colony PCR using DMSO, MangoTaq DNA Polymerase (Bioline) and pENTR TOPO M13 or pDONR207 FW and RV primers. The confirmed colonies were grown in liquid LB medium before the plasmids were extracted with the Qiaprep Spin Miniprep Kit (Qiagen). Plasmid DNA was sequenced using the Big Dye v3.1 protocol ahead of destination cloning.

In order to create expression vectors for all five genes, pENTR TOPO or pDONR207 vectors containing an ORF without the stop codon were recombined with the pUBC-GFP-DEST destination vector [22] using LR Clonase II Enzyme Mix (Invitrogen) to create pUB10:ORF:C-GFP constructs. Similarly, entry vectors containing a full ORF were recombined with a destination vector carrying the Cauliflower Mosaic Virus (CaMV) 35S promoter to create 35S:ORF constructs. In addition, pDONR207 vectors comprising promoter sequences were recombined with the pMDC163 destination vector carrying an ORF of the β-glucuronidase (GUS) reporter gene to create prom:GUS constructs [23]. DH5α *E*. *coli* cells were transformed as described above, grown overnight on selective media and confirmed by colony PCR using promoter and ORF-specific primers relevant to the different expression constructs. Plasmid DNA was retrieved as mentioned earlier and sequenced using the Big Dye v3.1 protocol prior to *Agrobacterium tumefaciens* transformation.

### Plant transformation and selection

Agrobacterium strain GV3101 [24] was transformed with the created expression vectors using a freeze/thaw method [25]. Subsequently, Agrobacterium colonies were selected with either 100 μg ml^-1^ spectinomycin (for pUB10:ORF:C-GFP and 35S:ORF constructs) or 50 μg ml^-1^ kanamycin (for prom:GUS constructs). Positive colonies were confirmed with colony PCR using primers relevant to the different expression vectors. Following this, Arabidopsis T0 plants were transformed by the inoculation of flower buds with Agrobacterium cell suspensions [26].

T1 seed lots generated from these transformations were screened for positive T1 plants by selection on 0.5x MS agar plates [27] with 0.1% (w/v) sucrose and 10 μg ml^-1^ hygromycin (for prom:GUS lines) or on soil with 20 μg ml^-1^ BASTA (glufosinate ammonium) spray solution (for pUB10:ORF:C-GFP and 35S:ORF lines). Afterwards, T2 seed lots produced by positive T1 plant transformants were subjected to the same selective treatments as used for the T1 generation. The potentially heterozygous and homozygous T2 plants that remained after screening the segregating T2 populations in this way were shown by PCR to carry the relevant transgenes.

Transgene overexpression driven by the 35S promoter was confirmed in comparison to wildtype by semi-quantitative RT-PCR (OneStep RT-PCR Kit, Qiagen) using primers relevant to the particular ORF and with alpha-(α)-Tubulin as a constitutive control [21]. With respect to the 35S line overexpressing the At1g74450 ORF, no single insertion homozygous T3 plants were identified based on BASTA resistance ratio analyses, an outcome which is discussed in more detail in the results section. Subsequent phenotypical analyses were therefore done with segregating T2 populations screened for positive transformants.

### Plant growth for phenotypical analyses

For plant growth on agar plates, seeds from the wildtype Col-0 and knockout mutant lines were sterilised by soaking them for 15 minutes in a 40% (v/v) bleach solution with a drop of Tween 20 (polyoxyethylenesorbitanmonolaureate). Sterilised seeds were planted on vertical 0.5x MS agar plates with Gamborg vitamins, 0.5 g/L MES buffer, 0.6% (w/v) sucrose, 1.3% (w/v) agar and a range of additives such as NaCl, MgSO_4_, ABA, JA, IAA and GA. For plant growth on soil, seeds from Col-0, knockout and 35S:ORF lines were stratified for three days at 4°C and then sown on Erin Multipurpose compost (Erin Horticulture; http://www.erinhorticulture.com) mixed with vermiculite (50:50). Stratified agar plates and soil trays were placed in controlled environment rooms under long days (16/8-h light/dark cycle) at 22°C and with approximately 50–60% relative humidity.

Knockout and overexpressing line populations grown on soil were compared to wildtype Col-0 weekly during the different stages of development from germination to senescence. Observed differences in plant height between populations of At1g74450 35S:ORF lines and wildtype Col-0 measured on day 37 following transfer of soil trays to controlled environment rooms were analysed statistically using one-way ANOVA followed by post hoc t-tests (two-tailed distribution, two-sample unequal variance) and Bonferroni correction for multiple testing (StatPlus:mac LE and Excel 2011).

### GUS-staining and imaging

GUS staining was performed as described previously [28]. Briefly, Arabidopsis flowers of the At1g74450 prom:GUS line were stained overnight at 37°C in 1 ml staining solution (1 mg ml^-1^ 5-bromo-4-chloro-3-indolyl-β-d-glucuronide, 100 mM sodium phosphate, pH 7.0, 10 mM Tris, pH 8.0, 1 mM EDTA, 0.05% Trition X-100, 5 mM potassium ferrocyanide, 5 mM potassium ferricyanide). Samples were destained with 70% ethanol at 37°C prior to observation. Images were collected using bright field on a Leica M165C microscope in combination with a MicroPublisher 5.0 RTV camera (QImaging).

### Confocal microscopy

Seeds of the At1g74450 pUB10:ORF:C-GFP line were sterilized and sown on 0.5x MS agar plates with 1% (w/v) sucrose, 0.5 g/L MES and 0.8% (w/v) agar and stratified for three days at 4°C. Seedlings were grown vertically for four to six days in a controlled environmental room as described above. For mitochondrial visualization, 6-day-old seedlings were incubated in 500 nM MitoTracker Orange (Life Technologies) in liquid growth medium for 15 minutes. Seedlings were rinsed in dye free media and mounted on a glass slide. For FM4-64 or FM4-64 + BFA visualization, 4-day-old seedlings were incubated in liquid growth medium with 2 μM FM4-64 (Life Technologies) or 2 μM FM4-64 + 50 μM BFA (Sigma Aldrich) for 30 and 60 minutes respectively. Fluorescence images of the epidermal cells at the root apical meristem and transition zone were collected on a Leica TCS SP5 confocal laser scanning upright microscope DM6000 using a 63x 1.2 NA HC PL Apo CS2 objective and equipped with Argon and HeNe lasers. Dual colour imaging for GFP and MitoTracker Orange was simultaneously acquired using the Argon 488 nm and HeNe 543 nm excitation lasers and emission collected at 495–530 nm and 560–624 nm respectively. Dual colour imaging for GFP and FM4-64 or GFP and FM4-64 + BFA was simultaneously acquired using the Argon 488 nm and HeNe 543 nm excitation lasers and emission collected at 500–530 nm and 650–800 nm respectively. Images were processed with the FIJI version of ImageJ (http://fiji.sc/Fiji) and further edited using Adobe Photoshop.

### Alexander staining

Anthers were dissected from flowers stage 12–13 according to the floral development stages described previously [29]. The flower buds, which at these stages were just opening, were carefully dissected under a dissecting microscope and anthers were placed on microscope slides in 2–4 drops of Alexander staining solution [30]. Cover slips were placed gently over the sample and sealed with VectaMountTM AQ (Vector Laboratories, USA). Slides were kept for 20 minutes at 37°C and examined using bright field on a Leica DMRB microscope. Micrographs were taken using a MicroPublisher 5.0 RTV digital camera (QImaging) and edited with Adobe Photoshop.

### Ruthenium red staining

Seeds were hydrated in 50 mM EDTA (pH 8.0) for 2 hours on a shaker at 250 rpm and room temperature [31]. After removal of EDTA, a 0.01% solution of ruthenium red was added to the seeds. Seeds were again placed on a shaker for 1 hour at 250 rpm and room temperature. Following ruthenium red staining, the seeds were washed with deionized water and examined using bright field on a Leica M165C microscope. Micrographs were taken using a MicroPublisher 5.0 RTV digital camera (QImaging) and edited with Adobe Photoshop. Surface areas of the mucilage layers were quantified using the FIJI version of ImageJ (http://fiji.sc/Fiji). Differences in mucilage layer surface area between populations of overexpressing lines and wildtype Col-0 were analysed statistically using one-way ANOVA followed by post hoc t-tests (two-tailed distribution, two-sample unequal variance) and Bonferroni correction for multiple testing (StatPlus:mac LE and Excel 2011).

## Results

### Homozygous knockout lines did not show altered macroscopic phenotypes

**Fig 1:**
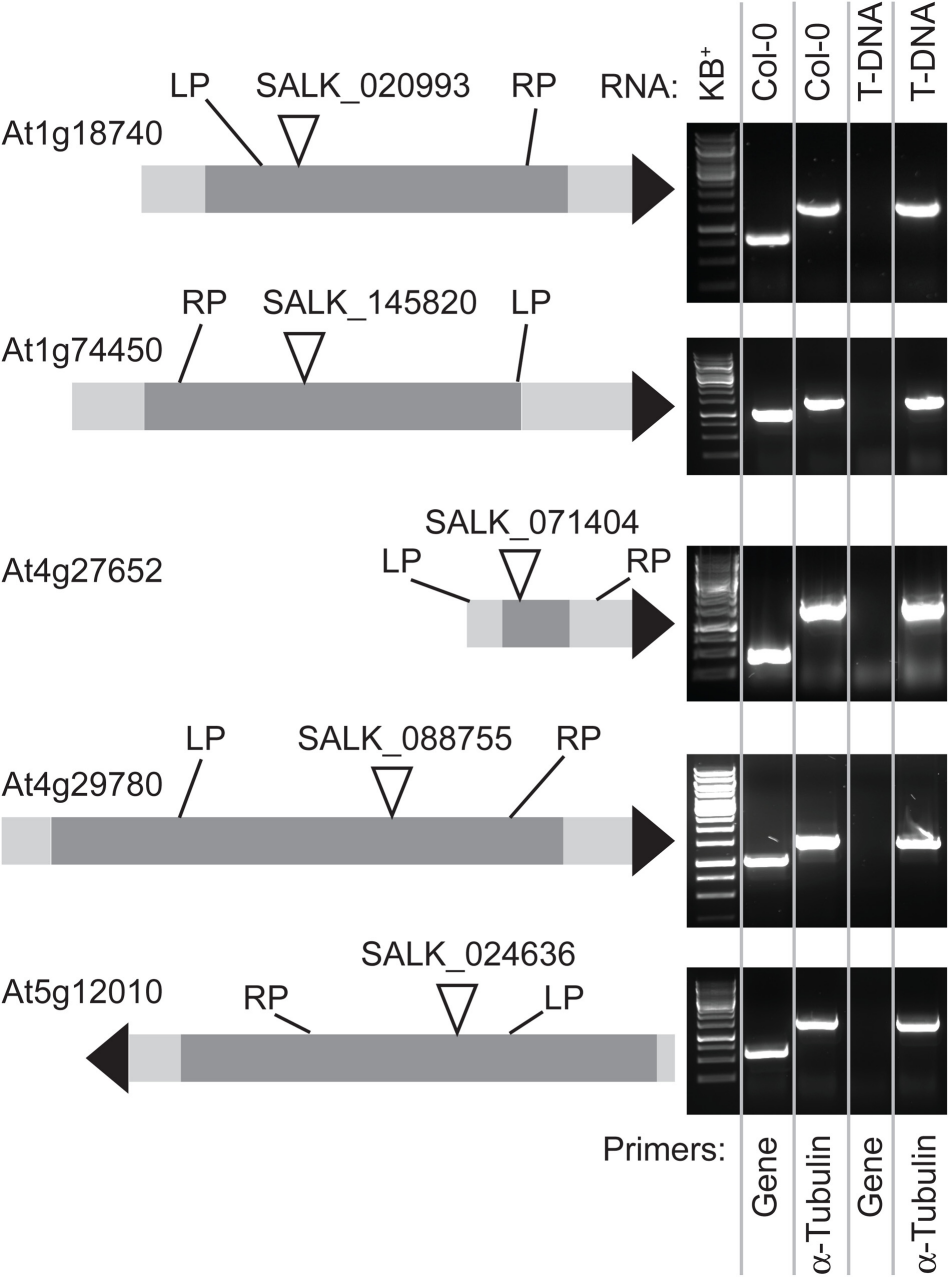
T-DNA insertion sites for five multiple-stress responsive genes. The diagram on the left shows the SALK T-DNA insertion sites for the At1g18740, At1g74450, At4g27652, At4g29780 and At5g12010 genes [20]. T-DNA insertion sites were obtained from the SIGnAL website (http://signal.salk.edu) whilst gene models were based on genomic DNA sequence information from the NCBI database. The gene models represent the mRNA (light grey) and protein coding (darker grey) regions of the five intronless genes. On the right, RT-PCR analyses using gene-specific primers (depicted as LP and RP relative to the T-DNA insertion in each gene model) show the loss of gene-specific mRNA transcripts in each homozygous, backcrossed T-DNA mutant, indicating that the selected five SALK lines are knockout lines. Alpha-(α)-Tubulin was used as a constitutive positive control with primers according to previous work [21]. Gene-specific RT-PCR primers are listed in S1 Table.

The aim of this study was to try to elucidate the functions of five multiple-stress responsive genes in Arabidopsis using reverse genetics techniques. Our first approach was to investigate whether gene knockout lines exhibit any phenotypical alterations compared to wildtype Col-0. SALK lines were selected that carried a T-DNA insertion in the protein coding region of each gene (Fig 1). The T-DNA lines were backcrossed to wildtype to eliminate multiple insertions and homozygous plants were subsequently assessed for the presence of relevant mRNA transcripts. RT-PCR results showed that the five homozygous T-DNA insertion lines are all knockout lines (Fig 1). Preliminary experiments indicated that knocking out gene At1g18740, At1g74450, At4g27652, At4g29780 or At5g12010 did not result in macroscopic phenotypical changes from wildtype under optimal growth conditions. In addition, exposure to several abiotic stress factors (NaCl, MgSO_4_, high light and drought) or plant hormones (ABA, Auxin, GA and JA) on vertical petri dishes and soil also did not lead to visible responses that were significantly different from wildtype.

### Overexpressing lines show few altered macroscopic phenotypes

**Fig 2:**
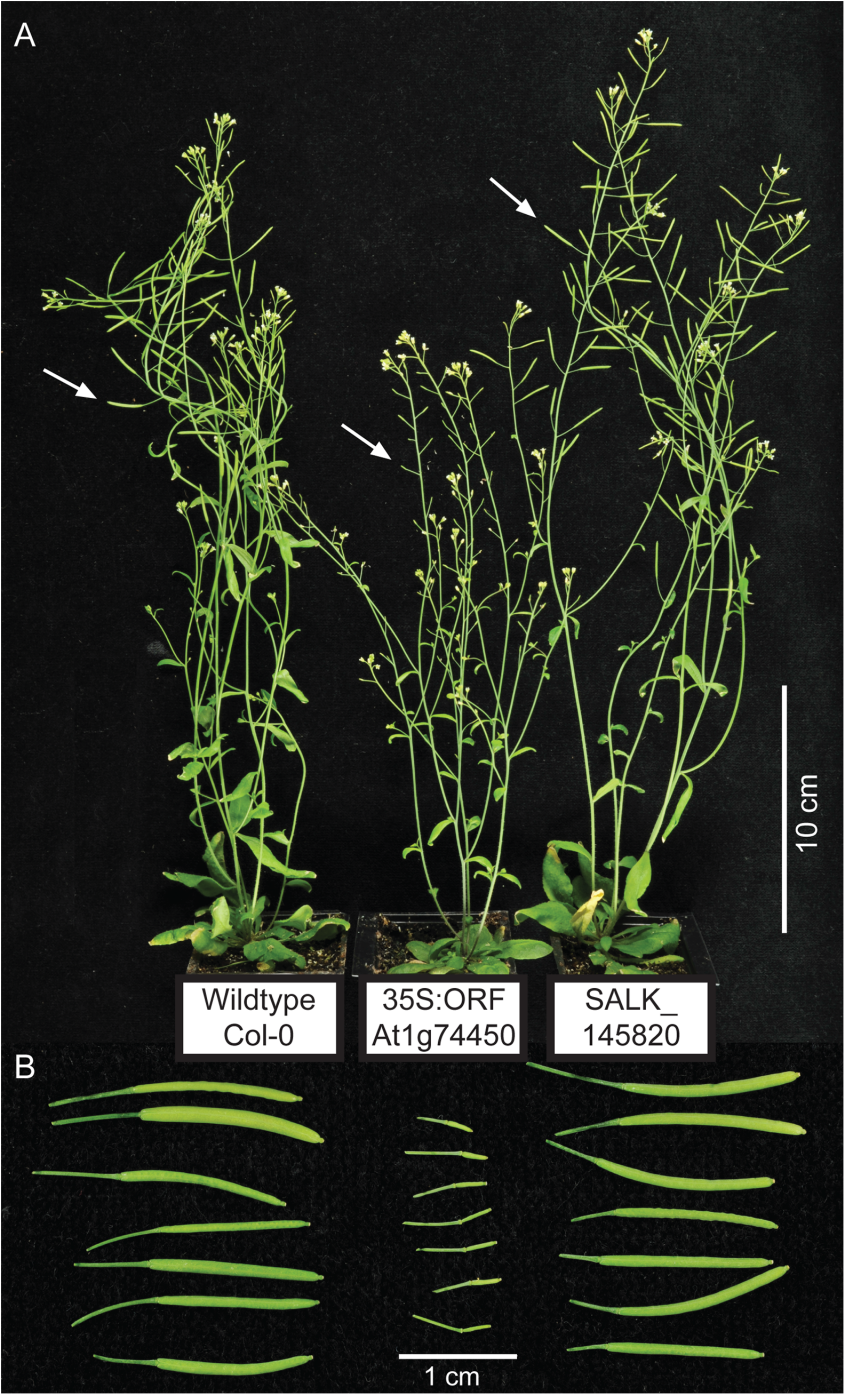
Altered phenotypes for the transgenic lines overexpressing the At1g74450 open reading frame. (A) Image showing a wildtype Col-0 plant on the left, a transgenic plant overexpressing the At1g74450 open reading frame (35S:ORF) in the middle and a SALK_145820 homozygous knockout plant for gene At1g74450 on the right. Arrows point to individual siliques. (B) Image of siliques corresponding to the plants shown in panel A.

Given the preliminary results from our knockout line analyses, our second approach was to create transgenic lines overexpressing gene-specific open reading frames. Overexpression lines driven by the 35S promoter were created for each gene and phenotypically analysed under optimal growth conditions. The 35S:ORF lines for genes At1g18740, At4g27652, At4g29780 and At5g12010 did not show macroscopic phenotypical differences from wildtype Col-0 during visual inspection. However, future studies addressing different aspects of plant stress response, growth or development may discover significant phenotypical alterations upon loss or overexpression of these genes that are beyond the scope of this current study. The only macroscopic phenotypes observed in this study were for the overexpressing line of the At1g74450 open reading frame (Fig 2). Therefore, the rest of the results section will mainly focus on findings related to the At1g74450 35S:ORF line. In addition, the homozygous knockout line for At1g74450 (SALK_145820) will be described for phenotypical comparison.

### Plants overexpressing the At1g74450 ORF show a shorter plant height phenotype

**Fig 3:**
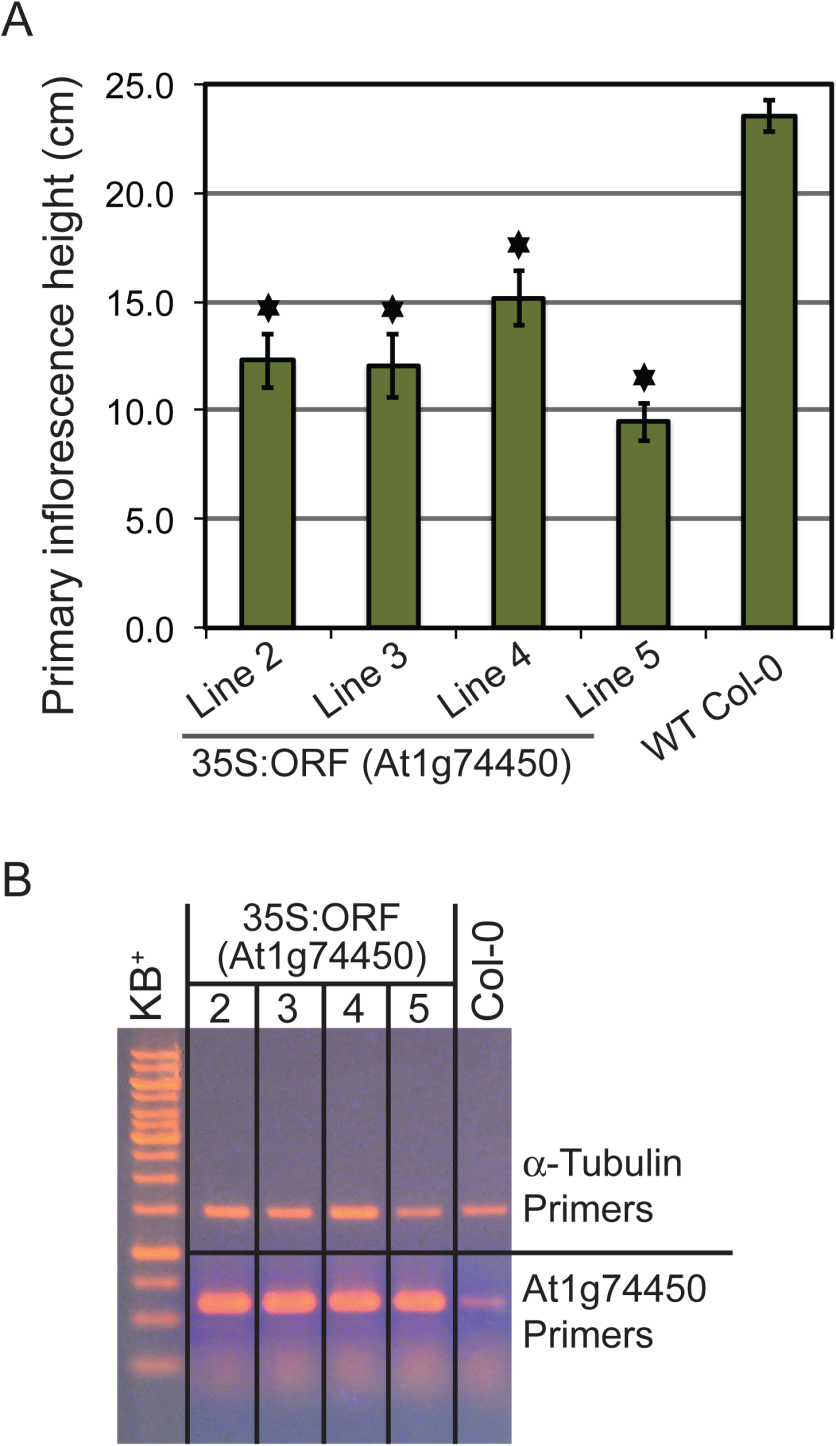
Primary inflorescence height of four independent transgenic lines overexpressing the At1g74450 open reading frame. (A) Graph showing the average primary inflorescence height in cm for four independent 35S:ORF lines compared to wildtype Col-0. Bars indicate standard error for each line (2: n = 31, 3: n = 27, 4: n = 30, 5: n = 20, Col-0: n = 117). The asterisks indicate statistically significant differences (p<0.01) between each 35S:ORF line and wildtype Col-0 based on one-way ANOVA followed by post hoc t-tests (two-tailed distribution, two-sample unequal variance) and Bonferroni correction. (B) Composite gel image showing semi-quantitative RT-PCR results for the four independent 35S:ORF lines and wildtype Col-0. The top half of the gel image shows RT-PCR products after 28 cycles generated by constitutive control Alpha-(α)-Tubulin primers [21]. The lower half of the gel image shows RT-PCR products after 28 cycles generated by gene-specific primers.

With respect to the transgenic line overexpressing the At1g74450 ORF, six primary transformants (T1) were recovered, four of which exhibited strong and similar phenotypes. Subsequently, an attempt was made to identify homozygous T3 plants. After identification of single-insertion T1 plants by analysing the segregation of T2 seeds on selective media, the intention was to harvest T3 seeds from up to eight T2 plants derived from each single-insertion T1 plant. Because most T2 plants showed a silique phenotype where no seeds were produced, in this particular instance, seeds could only be harvested from one normal looking and seed producing plant per T2 population. Ten T3 seeds derived from each T2 plant were plated on selective media and analysed for homozygous segregation. None of the seed-producing T2 plants seemed homozygous based on this analysis, and upon checking with PCR, none of the plants grown from the resulting T3 seeds carried the transgene insertion, although they showed resistance to BASTA and therefore presumably still carried the resistance gene. The results described in this manuscript are therefore based on the analysis of four T2 populations derived from four independent T1 plants that produced some seed later in development. The segregating T2 populations were screened for positive transformants using PCR and semi-quantitative RT-PCR (Fig 3B).

Fig 2A shows a 35S:ORF T2 plant that is shorter in height than either the flanking wildtype Col-0 or the At1g74450 knockout plant of the same age. To confirm that the shorter height of the overexpressing plants is significantly different from wildtype, the primary inflorescence height was measured for four T2 populations derived from four independent T1 plants and compared to Col-0 (Fig 3A). Statistical analysis using one-way ANOVA followed by post hoc t-tests and Bonferroni correction confirmed that the average primary inflorescence height of each overexpression line was significantly lower than the average height of wildtype.

### Lines overexpressing the At1g74450 ORF show reduced male fertility

**Fig 4:**
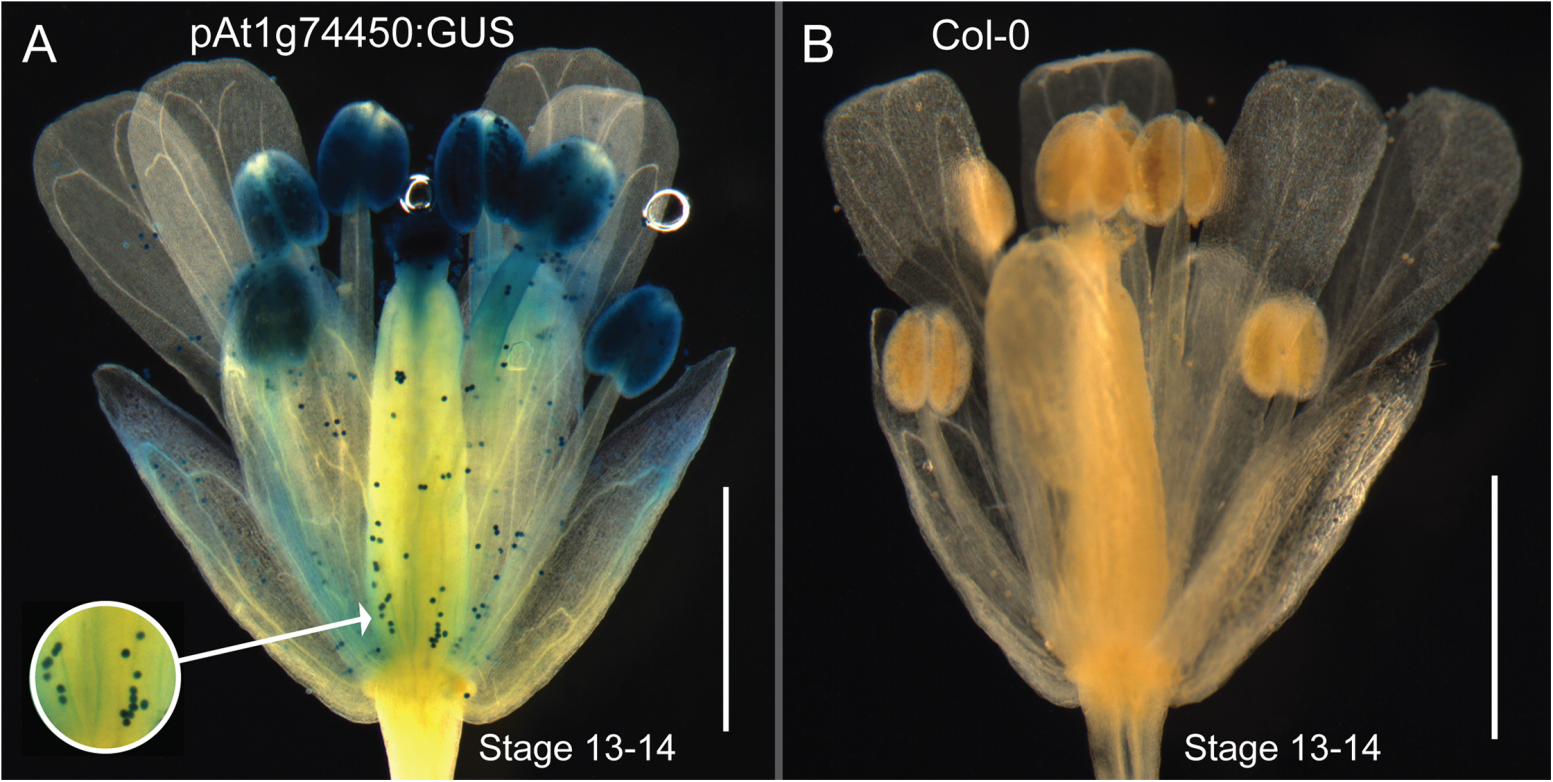
Localisation of GUS activity in pollen under the control of the At1g74450 promoter. (A) Flower of prom:GUS line at stage 13–14 showing GUS expression in pollen after treatment with staining solution. (B) Flower of wildtype Col-0 at stage 13–14 showing no GUS activity after treatment with staining solution. Scale bar represents 1 mm.

Fig 2A shows a 35S:ORF plant that has short, undeveloped siliques compared to wildtype and knockout plants of the same age. The siliques of the 35S:ORF plant are 2 to 3 mm in length and do not contain any seeds (Fig 2B). When fertilizing 35S:ORF flowers using wildtype Col-0 pollen, the siliques grew normally with regular numbers of seeds, suggesting that the silique phenotype is due to a male factor fertility problem. Interestingly, analysis of large-scale transcriptomic data indicates that At1g74450 is most highly expressed in mature pollen [17–19]. Expression of At1g74450 in pollen was confirmed *in planta* through analysis of our prom:GUS line, which detected the presence of GUS activity under the control of the At1g74450 promoter in pollen of Arabidopsis flowers (Fig 4A).

**Fig 5:**
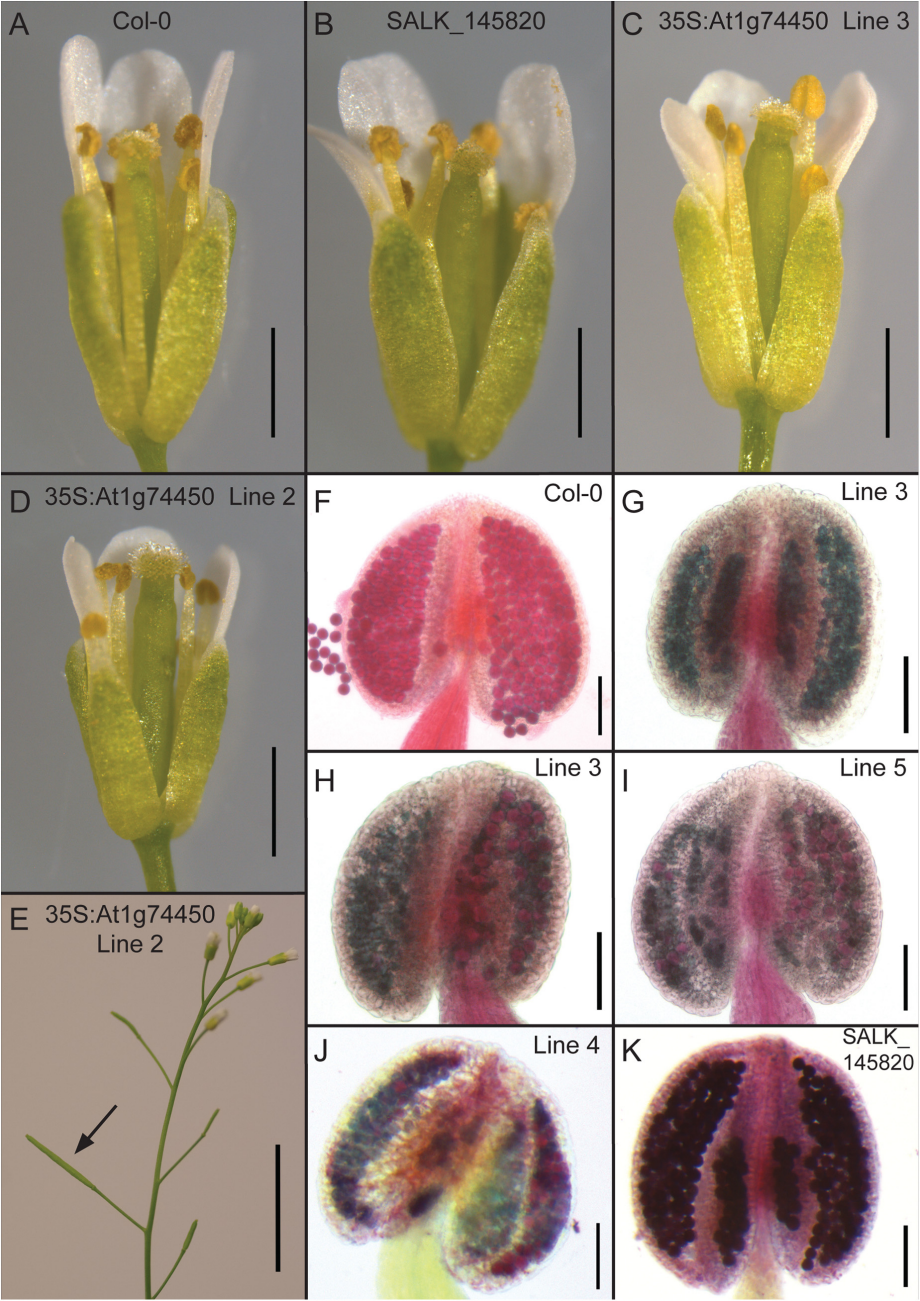
Phenotypical analysis of flowers and anthers from transgenic lines overexpressing the At1g74450 open reading frame. (A-D) Flowers at developmental stages 13–14. (A) Flower from wildtype Col-0 showing stamens with pollen. (B) Flower from SALK_145820 homozygous knockout line for gene At1g74450 showing stamens with pollen. (C) Flower from 35S:ORF line 3 showing stamens with no pollen. (D) Flower from 35S:ORF line 2 showing stamens with some pollen. (E) Plant from 35S:ORF line 2 showing one elongated silique flanked by undeveloped short siliques. (F-K) Anthers dissected from flowers stage 12–13 and treated with Alexander stain. (F) Anther from wildtype Col-0 showing viable pollen. (G) Anther from 35S:ORF line 3 showing no viable pollen. (H) Anther from 35S:ORF line 3 showing mostly non-viable and some viable pollen. (I) Anther from 35S:ORF line 5 showing mostly non-viable and some viable pollen. (J) Anther from 35S:ORF line 4 showing non-viable and viable pollen. (K) Anther from SALK_145820 homozygous knockout line for gene At1g74450 showing viable pollen. Scale bars represent 1 mm (A-D), 1 cm (E) and 100 μm (F-K).

Close-up investigation of Arabidopsis flowers at developmental stage 13–14 showed the presence of released pollen in flowers from wildtype Col-0 and At1g74450 knockout lines (Fig 5A and 5B). However, in some flowers of four independent overexpressing lines, a complete absence of pollen was observed when anthesis should have happened (Fig 5C). In other flowers, a reduced amount of released pollen was visible (Fig 5D).

Alexander staining of anthers from flowers at developmental stage 12–13 showed viable pollen for anthers from wildtype Col-0 and At1g74450 knockout lines (Fig 5F and 5K). For some anthers of the four 35S:ORF lines, no viable pollen was observed (Fig 5G), whilst for others, both non-viable and viable pollen were detected (Fig 5H–5J). The presence of some viable pollen could explain the observation that in some instances, particularly in late development, T1 and T2 plants from the four overexpressing lines carried one or several normally developed siliques (Fig 5E).

### Arabidopsis At1g74450:GFP fusion protein localizes to the cytosol *in vivo*

**Fig 6:**
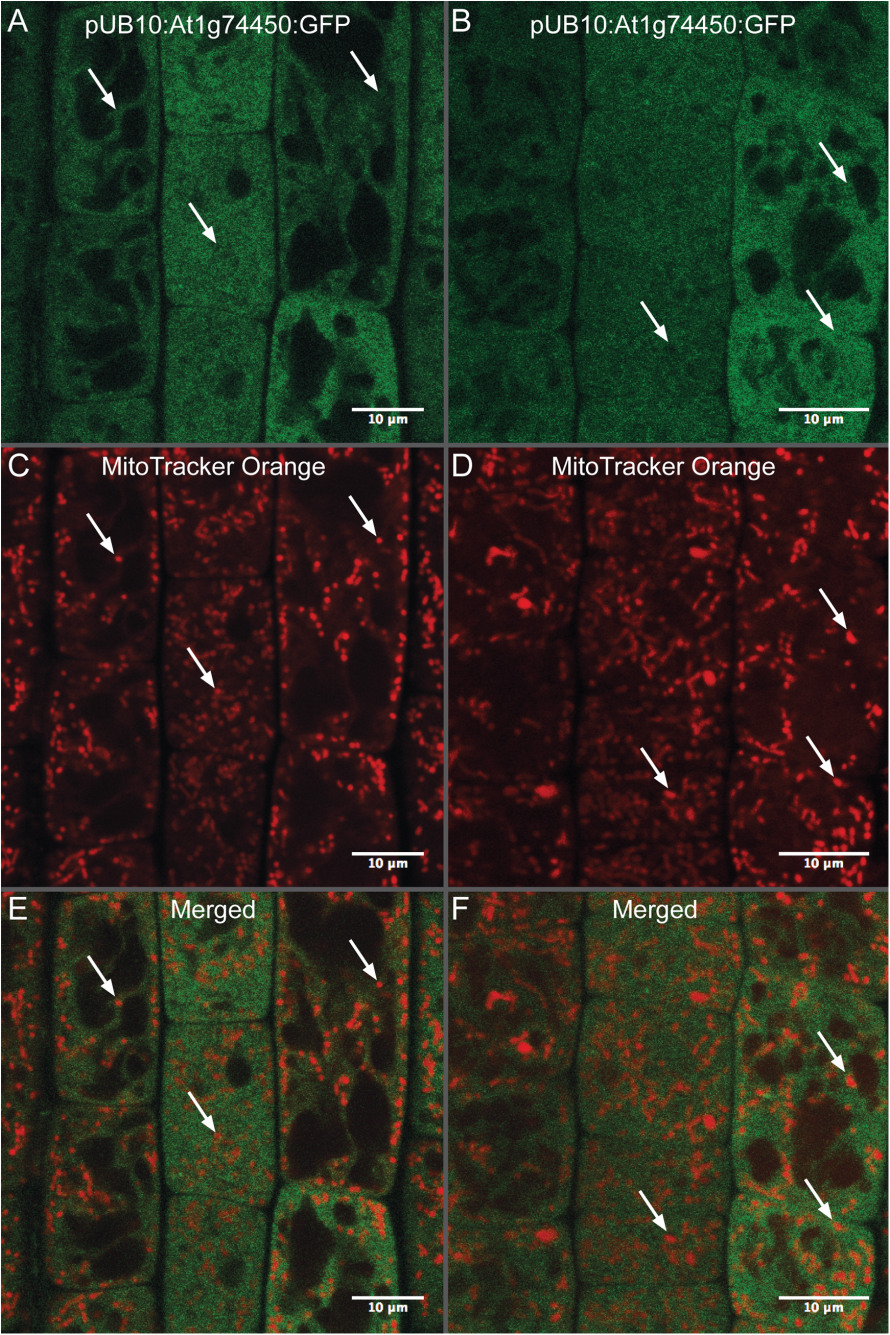
Localisation of GFP in epidermal cells at the root apical meristem and transition zone. (A-F) Confocal images of the transgenic line expressing the At1g74450 open reading frame fused with C-terminal GFP and driven by the ubiquitin-10 promoter (pUB10:ORF:C-GFP). (A, C and E) Images showing the same cells in one location of the root. (B, D and F) Images showing cells in a second location of the root. (A and B) GFP fluorescence is localised to the cytosol of root cells in two locations of the root. (C and D) Staining of mitochondria using MitoTracker Orange in two locations of the root. (E and F) Composite images showing no co-localisation of GFP signal with mitochondria when merging either panels A and C (E) or panels B and D (F). Scale bars represent 10 μm.

The predicted localisation of the At1g74450 protein varies across several sequence analysis tools available for protein localisation prediction. For example, the NCBI Gene database lists At1g74450’s cellular component as the nucleus, information which is inferred from sequence models. Sequence analysis by TargetP software predicts the presence of a mitochondrial targeting peptide (mtTP) with a reliability class (RC) value of 3 [32]. The presence of an mtTP would allow the At1g74450 protein to be imported into the mitochondrion. For *in planta* analysis of At1g74450’s sub-cellular localisation, we took confocal images of our transgenic line expressing the At1g74450 open reading frame fused with C-terminal GFP and driven by the ubiquitin-10 promoter (pUB10:ORF:C-GFP). GFP fluorescence images shown in Fig 6A and 6B indicate that the At1g74450 ORF:GFP fusion protein is present in the cytosol of cells in the root apical meristem and transition zone. Although the native At1g74450 gene is most highly expressed in pollen, roots show moderately high expression patterns compared to other tissues and were therefore selected for the localisation experiments [7,19]. Preliminary imaging of shoot tissues revealed a localisation similar to roots. Highlighting mitochondria with MitoTracker Orange dye and merging the GFP and MitoTracker images confirmed that the fusion protein did not get imported to mitochondria (Fig 6C–6F). However, these results do not exclude the possibility that the At1g74450 protein without a C-terminal tag does localize to mitochondria. Finally, sequence analysis by SignalP 4.1 software did not find evidence of a signal peptide cleavage site in the amino acid sequence of At1g74450 [32].

### At1g74450 shows similarity to sequences in Arabidopsis and other species

**Fig 7:**
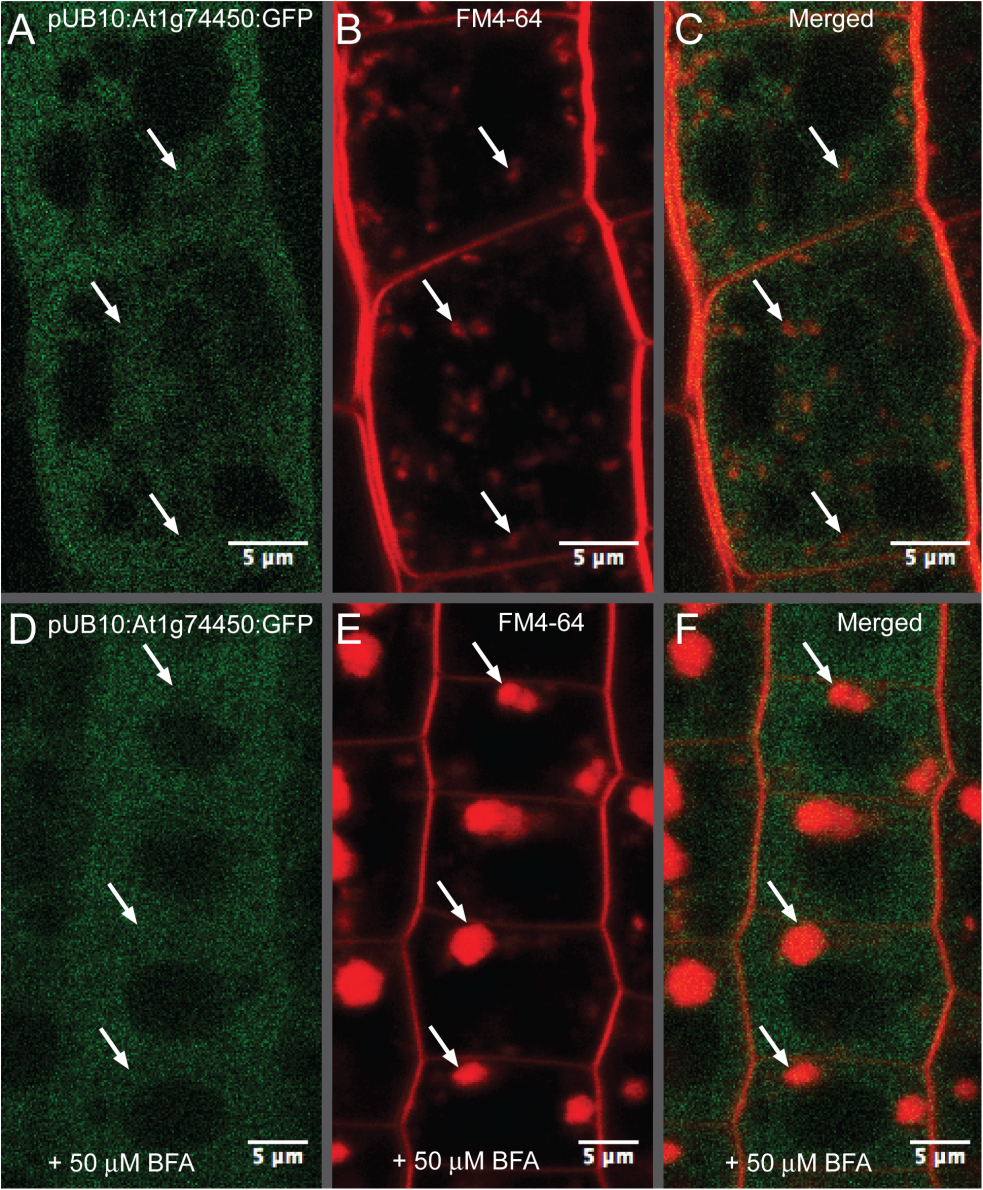
Analysis of At1g74450 protein co-localisation with FM4-64 or FM4-64 + BFA. (A-F) Confocal images of the transgenic line expressing the At1g74450 open reading frame fused with C-terminal GFP and driven by the ubiquitin-10 promoter (pUB10:ORF:C-GFP). (A and D) GFP fluorescence localisation. (B) 2 μM FM4-64 staining after 30 min. (E) 2 μM FM4-64 + 50 μM BFA staining after 1 hour. (C) Composite image showing no co-localisation of GFP signal with FM4-64 when merging panels A and B. (F) Composite image showing no co-localisation of GFP signal with FM4-64 + BFA when merging panels D and E. Scale bars represent 10 μm.

The At1g74450 protein sequence has two conserved protein domains: DUF793, which is a domain of unknown function and IPR008511, which refers to InterPro domain “Protein BYPASS related”. The Arabidopsis family of DUF793 proteins includes 11 members, of which *BYPASS1* and *ROH1* are the only functionally characterized genes to date [33]. A phylogenetic tree constructed using both unrooted neighbour-joining (NJ) and maximum likelihood (ML) methods placed At1g74450 in the same subfamily of DUF793-containing proteins as ROH1 [33]. Because it was found that ROH1 interacts with exocyst subunit Exo70A1 and potentially acts as an inhibitor of secretion [33], we tested whether the related At1g74450 protein is indeed cytosolic and does not localise to organelles of the endocytic and secretory pathways, such as for example the trans-Golgi network. Fig 7 shows that the GFP signal from the At1g74450 pUB10:ORF:C-GFP lines is not co-localised with the FM4-64 or FM4-64 + BFA signals, therefore excluding trans-Golgi network localisation. However, not all plant tissue types were tested. In addition, it is possible that the C-terminal GFP tag interferes with protein function, as the pUB10:ORF:C-GFP lines did not show phenotypes of reduced plant height and male fertility similar to the 35S:ORF lines. Furthermore, UB10-driven gene expression is known to be moderate compared to 35S [34] and therefore possible interactions with the trans-Golgi network may occur only at expression levels generated by the 35S promoter.

**Fig 8:**
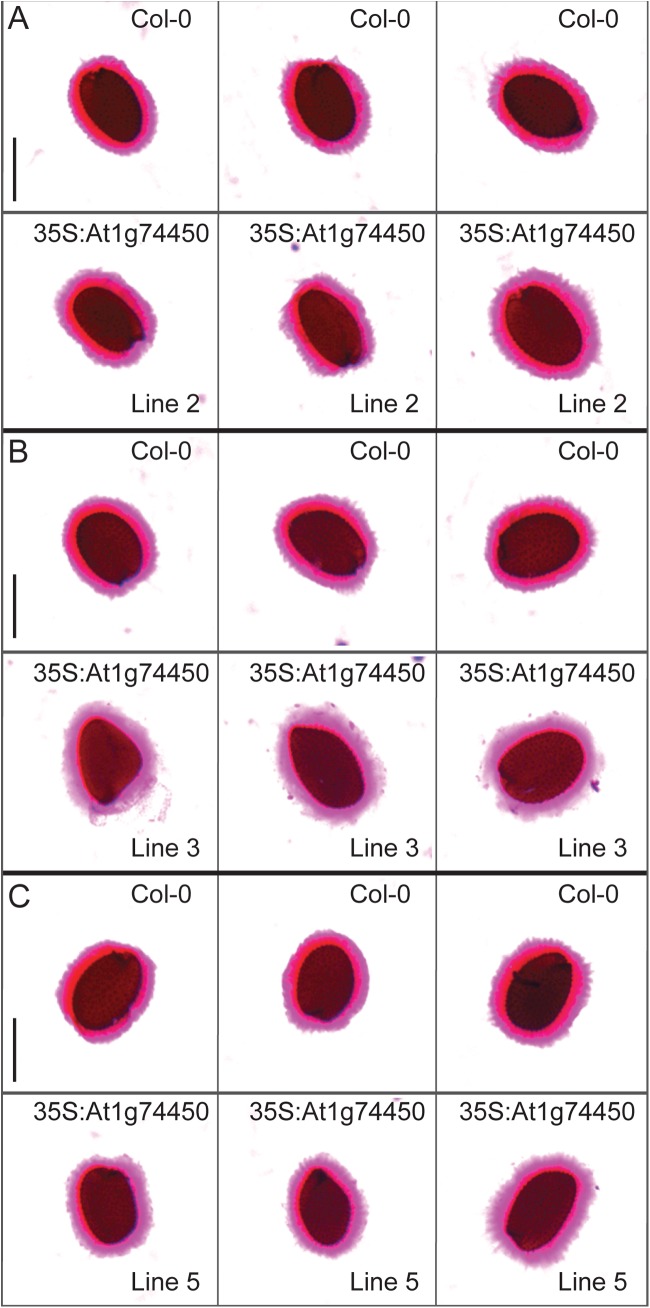
Ruthenium red-stained inner seed coat mucilage layer after imbibition of wildtype and At1g74450 35S:ORF seeds. (A) Wildtype Arabidopsis ecotype Columbia-0 (top row) and At1g74450 35S:ORF line 2 (bottom row). (B) Col-0 (top row) and 35S:ORF line 3 (bottom row). (C) Col-0 (top row) and 35S:ORF line 5 (bottom row). Scale bars represent 500 μm.

**Fig 9:**
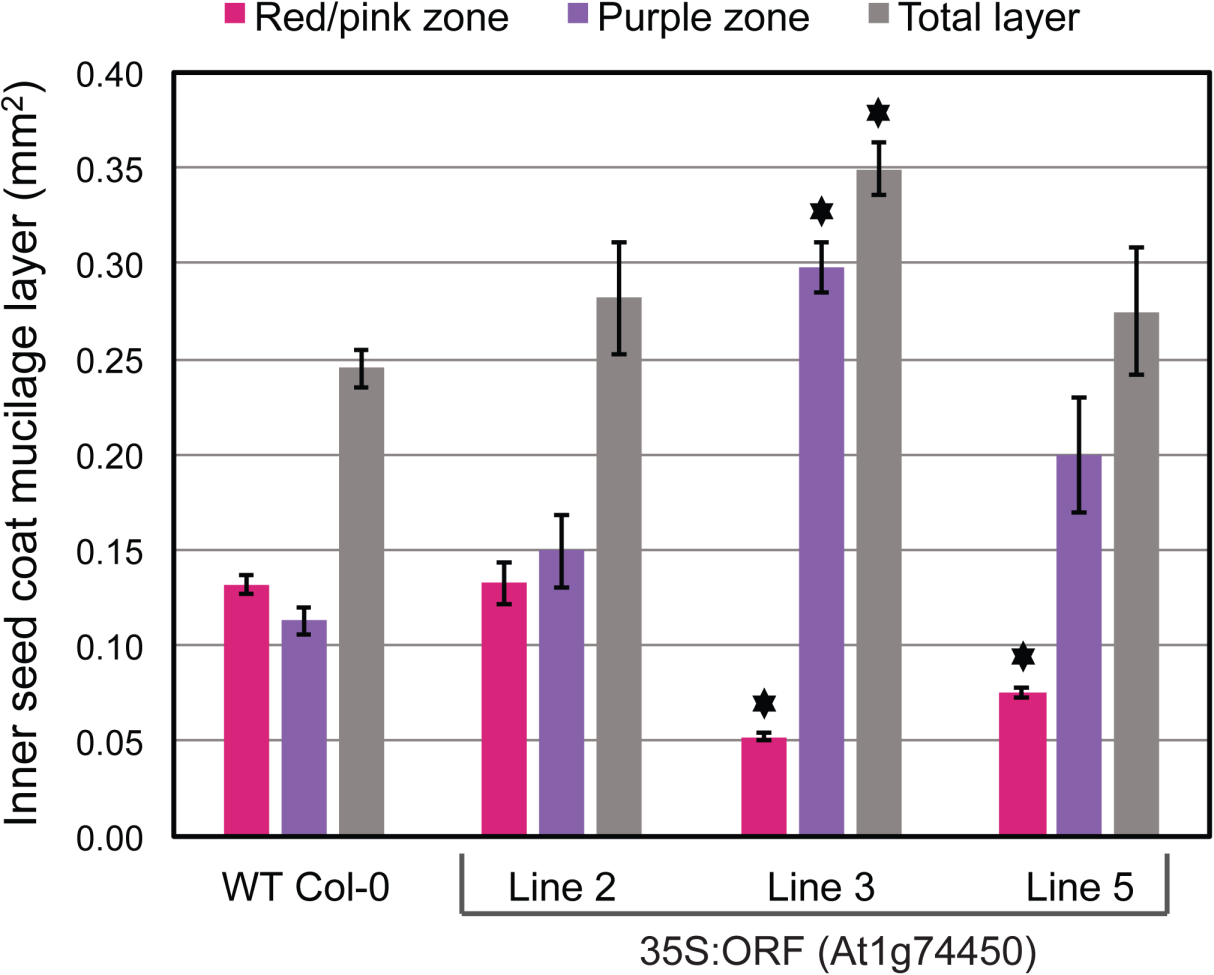
Quantification of the inner seed coat mucilage layer depicted in Fig 8. Graph showing the average surface areas in mm^2^ of the red/pink zone, the purple zone and the combined total layer for three independent At1g74450 35S:ORF lines compared to wildtype Col-0. Bars indicate standard error for each line (2: n = 3, 3: n = 3, 5: n = 3, Col-0: n = 9). The asterisks indicate statistically significant differences (p<0.01) between each 35S:ORF line and wildtype Col-0 based on one-way ANOVA followed by post hoc t-tests (two-tailed distribution, two-sample unequal variance) and Bonferroni correction.

When studying the At1g74450 35S:ORF lines, analysis of the inner seed coat mucilage layer revealed that, unlike for *ROH1*, overexpression of the At1g74450 gene does not reduce the amount of mucilage deposition (Figs 8 and 9). Although the total surface area of the inner mucilage layer is not smaller than that of wildtype (Fig 9), 35S:ORF lines 3 and 5 in particular show variations in staining (Fig 8B and 8C). Quantification of the red/pink and purple staining zones depicted in Fig 8 reveals that lines 3 and 5 have a significantly smaller red/pink zone than wildtype Col-0 (Fig 9). This may indicate that overexpressing At1g74450 affects the composition of the inner seed coat mucilage layer [35–37]. Overall, results from the overexpression lines indicate that the related At1g74450 and ROH1 proteins carrying DUF793 domains affect seed coat mucilage differently.

NCBI protein BLAST (blastp) results for the At1g74450 amino acid sequence using NCBI Reference Sequence NP_565086.1 indicate that the sequence is plant specific, but not lineage specific. Similar sequences are present throughout angiosperms (rosids, asterids, monocots) and in *Amborella trichopoda*. However, no relatives are detected in algae [33]. The At1g74450 protein shows high similarity to sequences of related Brassicaceae species such as *Arabidopsis lyrata* (95%), *Capsella rubella* (91%) and *Eutrema salsugineum* (84%). In addition, it is 70% identical to At1g18740, another multiple-stress responsive gene in Arabidopsis containing the DUF793 domain. As mentioned earlier, preliminary experiments with knockout and overexpression lines for the At1g18740 gene did not show any altered phenotypes. Furthermore, a double knockout mutant for the related At1g18740 and At1g74450 genes showed no macroscopic differences from wildtype either.

## Discussion

Although less than half of Arabidopsis protein-coding genes have had some aspect of their functions annotated based on experimental evidence [8], this percentage seems to be higher for a subset of 197 multiple-stress responsive genes reported previously [6]. At the start of this study, only 35 out of 197 genes in the set remained uncharacterized with respect to their biological or molecular functions. From these 35 unknown genes, we selected five genes to investigate in detail using reverse genetics techniques. Preliminary experiments using knockout and overexpressing lines identified macroscopic phenotypes for one of them: At1g74450. However, our findings do not preclude the possibility that future efforts will reveal (microscopic or biochemical) phenotypes caused by the other four multiple-stress responsive genes that were included in this study.

The results presented for gene At1g74450 provide experimental evidence for some of the biological processes that its protein product is involved in. Phenotypical analyses of 35S-driven overexpressing lines showed that At1g74450 reduces plant height and male fertility: the latter by affecting pollen development. The absence of similar phenotypes in the UB10-driven GFP-tagged lines could be due to interference of the GFP tag with protein function or to differences between the promoters, with UB10-driven gene expression known to be moderate compared to 35S [34]. A review of the literature reveals several other genes known to affect both shoot and pollen development in Arabidopsis. For example, the Arabidopsis *echidna* (*ech*) T-DNA insertion mutant was shown to have a dwarf phenotype with a high level of branching [38]. In addition, the *ech* mutant exhibited significantly reduced fertility, although some normal silique elongation and seed set still occurred [39]. It was found that the ECHIDNA protein mediates trans-Golgi network trafficking associated with the delivery of cell wall components [38–40]. In the case of At1g74450 however, we have so far found no evidence of trans-Golgi network localisation or an involvement in the secretion of cell wall components, despite the fact that a role in seed coat pectin deposition was previously described for the related ROH1 gene [33].

In another study, T-DNA insertion mutants (*cs7*) of the *Callose Synthase 7* (*CalS7*) gene exhibited a moderate reduction in seedling height and produced aberrant pollen grains and short siliques with aborted embryos, suggesting that *CalS7* plays a role in plant growth and reproduction [41]. Although *CalS7*’s main function is associated with callose deposition in the phloem of vascular tissues, the fact that pollen grains of *cs7* mutants clumped together in a tetrad-like manner suggests that *CalS7* might have a minor role in the formation of the callose wall during pollen development or the release of microspores from tetrads [41].

The simultaneous silencing of *CCR1*, *CAD C* and *CAD D*, three Arabidopsis genes encoding two enzymes of the lignin biosynthetic pathway, also affects plant height and male fertility [42]. The triple mutant anthers actually contained viable pollen and developed normally, but they failed to dehisce and this led to a phenotype of male sterility [42]. Interestingly, the abiotic condition of Cu insufficiency is linked to shoot and pollen developmental defects through the *SPL7* and *KIN17* genes, with a double knockout mutant leading to growth arrest and pollen inviability, amongst other changes [43].

Focussing on the reduced male fertility phenotype in particular, we find several examples in the literature of molecular factors that potentially link stress factor exposure to pollen development without necessarily affecting shoot development. In general, recent research addressing the impact of environmental stress on male gametogenesis has revealed an important and often intertwined role for cytoskeletal dynamics, tapetal ER stability, sugar metabolism and oxidative stress, and has additionally demonstrated the putative involvement of several stress signalling components, including major plant hormones (ABA, GA and auxin) and epigenetic regulators such as smRNAs [44].

The tapetum especially has been shown to be critical for functional pollen formation, with many of the characterized male-sterile mutants exhibiting abnormal tapetal development [39]. In rice, ectopic persistence of the tapetal cell layer is thought to be the major factor underlying cold-induced pollen abortion and male sterility (at 19°C), similarly as observed in the tapetal programmed cell death defective *tdr* mutant [44]. The tapetal rough endoplasmic reticulum (RER) can change structurally following temperature stress and is known to facilitate the degradation of the callosic cell wall that surrounds developing microspores [45–47]. Our results suggest that the At1g74450:GFP fusion protein does not localize to the ER *in vivo*. Furthermore, sequence analysis by SignalP 4.1 software did not find evidence of an ER signal peptide cleavage site in the At1g74450 protein [32]. Future studies could investigate whether At1g74450 plays a role in tapetal development and whether tapetal ER instability may be a downstream effect of At1g74450 protein activities.

With respect to sugar metabolism, the expression of invertase genes in male reproductive tissues is known to be down-regulated by stress factors such as heat, cold and drought [48–52]. Complete silencing of a tomato invertase gene led to a significant reduction in pollen viability [53], indicating that invertase genes seem to be an important link between stress exposure and reduced male fertility. The potential role of At1g74450 in sugar metabolism is unclear and at most an indirect one, since blastp analysis of the At1g74450 protein did not show any sequence similarity to enzymes known to possess invertase or other relevant activities.

In general, there are several reports of stress responsive genes affecting pollen development without reducing overall male fertility. For instance, T-DNA insertion plants for the *AtREN1* gene, which contains a heat shock factor like domain, are defective in heat stress response and produce a notably higher proportion of aberrant pollen grains [54]. The authors found that the AtREN1 protein is targeted specifically to the nucleolus and that it is likely to be involved in ribosomal RNA biogenesis or other nucleolar functions. In another case it was shown that the pollen-specific transcription factor *WRKY34*, which mediates cold sensitivity of mature pollen, is required for male gametogenesis, where it functions redundantly with *WRKY2*. [55,56].

Non-genic molecular factors linked to both stress response and pollen development are, for example, reactive oxygen species (ROS) and several plant hormones. ROS are known to accumulate in plants in response to a wide range of different environmental stress factors. In transgenic rice plants, reducing the expression of the *MT-1-4b* gene, which is involved in ROS scavenging, caused an increased level of superoxide anion and a decrease in pollen fertility [57]. In Arabidopsis, a double mutant for the glutaredoxin genes *ROXY1* and *ROXY2* was shown to be sterile and unable to produce pollen [58]. Evidence suggests that the At1g74450 gene is up-regulated in response to reactive oxygen species such as singlet oxygen ^1^O_2_ [59,60]. In addition, it was found to be up-regulated in the *mkk1/2* double mutant, which accumulates ROS [61]. Mitogen-activated protein kinases MKK1 and MKK2 are part of a cascade that regulates ROS and salicylic acid (SA) accumulation. Although we know that the expression of the At1g74450 gene is up-regulated in response to ROS, the exact molecular roles of the At1g74450 protein as a negative regulator of male fertility remain to be investigated.

With respect to plant hormones, links between stress response and pollen development have so far been reported for elevated levels of ABA, as well as for reduced levels of auxin [44]. Analysis of transcriptome data suggests that the At1g74450 gene is highly up-regulated in response to exogenous application of methyl jasmonate (MeJA) [18]. Although it is known that jasmonate signalling is required for fertility, it is jasmonate deficiency that is generally associated with male-sterility in Arabidopsis [62]. Potential interactions between MeJA and At1g74450 in relation to our reported male fertility and plant height phenotypes are therefore unresolved and could be the focus of future research. Transcriptome data analysis also showed a significant up-regulation of At1g74450 in response to exogenous application of auxin [18]. However, it was found that tissue-specific auxin reduction, rather than accumulation, led to the abortion of pollen development in barley and Arabidopsis in response to high temperatures [63].

Brassinosteroids (BR) have been shown to affect both plant height and male fertility in Arabidopsis. For example, BR-deficient and BR-insensitive mutants of Arabidopsis are generally dwarfed with shorter petioles and hypocotyls [64]. In addition, many of these mutants also show negative effects on male fertility, such as reduced pollen number, viability, and release efficiency [65]. However, it is not clear whether endogenous BR levels function as a link between exposure to environmental stresses and shoot/pollen development. More research is needed to determine whether At1g74450 interacts with brassinosteroid biosynthesis and signalling. Finally, reduced endogenous levels of the plant hormone gibberellin (GA) lead to changes in plant height and fertility by affecting the accumulation of DELLA proteins. Environmental stress factors such as high salinity and cold both cause reductions in the levels of bioactive GAs in Arabidopsis, consequently leading to growth repression through the accumulation of DELLA proteins [66–68]. Conversely, increased GA biosynthesis and signalling promote growth in response to shading and submergence [69]. With respect to fertility, it was shown that the Arabidopsis DELLA proteins RGA and RGL2 jointly repress petal, stamen and anther development in GA-deficient plants, and that this function is enhanced by RGL1 activity [70]. Future research could assess whether the At1g74450 protein is involved in GA- and/or DELLA-dependent processes that result in growth inhibition and/or reduced male fertility.

Overall, it is clear that several genes in Arabidopsis are associated with a reduction in both plant height and male fertility, through a variety of mechanisms. Except for genes encoding DELLA proteins, none of them seem to provide a potential link between multiple stresses and shoot/pollen development similar to what is reported here for the overexpression of At1g74450. Analysis of large-scale transcriptome data indicates that the expression of gene At1g74450 is up-regulated in response to high light, cold, salt, drought, heat, hypoxia, selenate, magnesium sulphate, iron deficiency as well as biotic stress factors [7,18]. In general, *in planta* fluctuations of reactive oxygen species and hormones seem to be candidates for the up-regulation of the At1g74450 gene in response to multiple stress factors. With respect to the At1g74450 protein, further research needs to be done to analyse whether it shows interaction with any of the gene products, hormones or reactive oxygen species known to affect plant height negatively, impact pollen development, or both. Of particular interest is whether such interactions would occur at At1g74450 expression levels seen in response to multiple stresses in wildtype Col-0 tissues, such as pollen, as this information would be needed to rule out the possibility that the altered phenotypes caused by 35S-driven overexpression of the At1g74450 ORF are due to interactions that do not normally occur at up-regulated expression levels driven by the native At1g74450 promoter *in planta*.

Although it is apparent that the effects of stress on crop productivity are most severe at developmental stages such as male gametophyte development [5], the molecular factors and regulatory networks underlying environmental stress-induced male gametophytic alterations are still largely unknown in plants in general [44]. This study elucidates some of the potential functions of a previously uncharacterised gene in the model species *Arabidopsis thaliana*. The At1g74450 protein seems to be localised to the cytosol, as shown in root tips, and may play a role in shoot and pollen development. We are not aware of any other genes previously linked to shoot and male gametophyte development that are known to be up-regulated in response to many different environmental stresses. Future studies related to At1g74450 could focus on the discovery of its exact molecular functions (biochemical activities and interactions) and the exact expression levels, cell types and time points during development that are involved in its effects on shoot height and pollen viability. Due to high levels of homology with unknown genes in *Arabidopsis lyrata*, *Capsella rubella* and *Eutrema salsugineum*, the possibility exists that some functional similarity may be found in these sister species.

## Supporting Information

S1 Table Primer sequences. Overview of primers used for T-DNA knockout line confirmation and transgenic plant generation.
(PDF)
