# Supplementary material for: Overexpressing the Multiple-Stress Responsive Gene At1g74450 Reduces Plant Height and Male Fertility in Arabidopsis thaliana
Source: PLoS One. 2015 Oct 20;10(10):e0140368. doi: 10.1371/journal.pone.0140368 (PMC4619001; doi:10.1371/journal.pone.0140368)
Supplement: S1 Table — Overview of primers used for T-DNA knockout line confirmation and transgenic plant generation. (PDF) [file pone.0140368.s001.pdf]

S1 Table. Primer sequences. Overview of primers used for T-DNA knockout line confirmation and transgenic plant generation

Knockout mutant confirmation:

PCR:

Gene ID	Left genomic primer	Right genomic primer
At1g18740	ACCACTAGACGTGTCCACTGC	ACATCACAAAAAGCAAAACCG
At1g74450	AACCATGATGCTTTCCAACAG	CGAGTTGACTCAGATGGAAGC
At4g27652	AAACTGCGTATTAAACGTCGTG	ATCGTCAAATGAGCCATCATC
At4g29780	TACTCTGCAGCTGGTGAAACC	AGATCCTGAAGCTTCACCTCC
At5g12010	TCAACTTGCGTGTTCACACTC	ACATCCCCTTCAACAAACCTC

Gene ID	T-DNA left border primer LBb1.3	Right genomic primer
At1g18740	ATTTTGCCGATTTCGGAAC	ACATCACAAAAAGCAAAACCG
At1g74450	ATTTTGCCGATTTCGGAAC	CGAGTTGACTCAGATGGAAGC
At4g27652	ATTTTGCCGATTTCGGAAC	ATCGTCAAATGAGCCATCATC
At4g29780	ATTTTGCCGATTTCGGAAC	AGATCCTGAAGCTTCACCTCC
At5g12010	ATTTTGCCGATTTCGGAAC	ACATCCCCTTCAACAAACCTC

RT-PCR:

Gene ID	Left genomic primer	Right genomic primer
At1g18740	GCTTCTTCAAACGACCTTCTCT	TCACCTCAACTTCCTTGTCATC
At1g74450	AACCATGATGCTTTCCAACAG	CGAGTTGACTCAGATGGAAGC
At4g27652	CCACAACAACACCAATCACAC	AAAAATCTCAGCCGTCCAATC
At4g29780	TACTCTGCAGCTGGTGAAACC	AGATCCTGAAGCTTCACCTCC
At5g12010	GTGGGAAGAGTGTAGCCGATT	ACATCCCCTTCAACAAACCTC

Transgenic plant generation:

TOPO entry cloning (blunt-end):

Gene ID	primers for N-terminal fusion
At1g18740	ORF: FW: CACCATGCCAGCTACGGATTTTCAAG
	RV: TCAAAGAGAATCAAGACTCTCAG
At1g74450	FW: CACCATGCCAGCAACGGAATATCAAAG
	RV: TCATTCAGAACCATGATGCTTTCC

Gene ID	primers for C-terminal fusion
At1g18740	FW: CACCATGCCAGCTACGGATTTTCAAGG
	RV: AAGAGAATCAAGACTCTCAGTTCTG
At1g74450	FW: CACCATGCCAGCAACGGAATATCAAAG
	RV: TTCAGAACCATGATGCTTTCCAAC

Transgenic plant generation:

AttB entry cloning:

Gene ID	primers for N-terminal fusion
At4g27652	ORF: FW: GGGGACAAGTTTGTACAAAAAAGCAGGCTTCATGATCTCCGTCGTAATCA
	ORF: RV: GGGGACCACTTTGTACAAGAAAGCTGGGTCTCAGAAATCAACGAGATGAG
At4g29780	ORF: FW: GGGGACAAGTTTGTACAAAAAAGCAGGCTTCATGGAAATCTCTTCTTTCC
	ORF: RV: GGGGACCACTTTGTACAAGAAAGCTGGGTCCTATAGAGTTCTTGTCCCGG
At5g12010	ORF: FW: GGGGACAAGTTTGTACAAAAAAGCAGGCTTCATGAAAGCCGCCGTTTTCC
	ORF: RV: GGGGACCACTTTGTACAAGAAAGCTGGGTCTTATAGGAAAGAAGTACCCG

Gene ID	primers for C-terminal fusion
At1g18740	Prom: FW: GGGGACAAGTTTGTACAAAAAAGCAGGCTTCTCATGTTAGGAGCAATTGT
	Prom: RV: GGGGACCACTTTGTACAAGAAAGCTGGGTCCCTCAAAACAGATGCAAAGT
At1g74450	Prom: FW: GGGGACAAGTTTGTACAAAAAAGCAGGCTTCACTTTCTTGGAAAGACGAA
	Prom: RV: GGGGACCACTTTGTACAAGAAAGCTGGGTCCCTCAAAATGAAAACACACA
At4g27652	ORF: FW: GGGGACAAGTTTGTACAAAAAAGCAGGCTTCATGATCTCCGTCGTAATCA
	ORF: RV: GGGGACCACTTTGTACAAGAAAGCTGGGTCGAAATCAACGAGATGAGAAG
	Prom: FW: GGGGACAAGTTTGTACAAAAAAGCAGGCTTCTTACCAGATGTCAAAAGTC
	Prom: RV: GGGGACCACTTTGTACAAGAAAGCTGGGTCGATCGATTTATAAAGTTGGA
At4g29780	ORF: FW: GGGGACAAGTTTGTACAAAAAAGCAGGCTTCATGGAAATCTCTTCTTTCC
	ORF: RV: GGGGACCACTTTGTACAAGAAAGCTGGGTCTAGAGTTCTTGTCCCGGCAA
	Prom: FW: GGGGACAAGTTTGTACAAAAAAGCAGGCTTCAGAAGTTGATCGCTACTAC
	Prom: RV: GGGGACCACTTTGTACAAGAAAGCTGGGTCTGTTTCAGAGCTTTGACGAA
At5g12010	ORF: FW: GGGGACAAGTTTGTACAAAAAAGCAGGCTTCATGAAAGCCGCCGTTTTCC
	ORF: RV: GGGGACCACTTTGTACAAGAAAGCTGGGTCTAGGAAAGAAGTACCCGCGA
	Prom: FW: GGGGACAAGTTTGTACAAAAAAGCAGGCTTCTCTTCCACATCAAAGTTCT
	Prom: RV: GGGGACCACTTTGTACAAGAAAGCTGGGTCCGAAGTTGTGTTGTTTCACG
